# Using Andersen’s behavioral model of health care use for intermittent preventive treatment of malaria in pregnancy in Nigeria

**DOI:** 10.1186/s12884-023-05648-9

**Published:** 2023-05-04

**Authors:** Bola Lukman Solanke, Rasheed Adebayo Yinusa, Olaoye James Oyeleye, Omolayo Bukola Oluwatope, Benjamin Bukky Ilesanmi, Tosin Olajide Oni

**Affiliations:** 1 Department of Demography and Social Statistics, Obafemi Awolowo University, Ile-Ife, Nigeria; 2 Department of Demography and Social Statistics, Federal University, Birnin-Kebbi, Nigeria; 3 National Centre for Technology Management, Obafemi Awolowo University, Ile-Ife, Nigeria

**Keywords:** Malaria in pregnancy, IPTp, Andersen model, Childbearing women, Nigeria

## Abstract

**Background:**

Studies in Nigeria and elsewhere in sub-Saharan Africa (sSA) have explored factors influencing usage of intermittent preventive treatment of malaria in pregnancy (IPTp). Most studies, however, are not model or theory-based, which provides less satisfactory guidance to malaria control programming. This study fills the knowledge gap by adapting Andersen’s behavioral model of health care use to IPTp usage in Nigeria.

**Methods:**

This study adopted a cross-sectional design that utilized secondary data extracted from the 2018 Nigeria Demographic and Health Survey (NDHS). A weighted sample of 4,772 women who had given birth in the past year preceding the survey, was analyzed. Outcome variable was usage of IPTp, dichotomized into optimal or otherwise. Explanatory variables cut across individual and community levels and were divided into predisposing, enabling and need factors in line with the theoretical constructs of the Andersen model. Two multilevel mixed-effects logistic regression models were fitted to identify factors which influenced optimal usage of IPTp. Analyses were performed using STATA 14. Statistical significance was set at 5%.

**Results:**

Realised level of optimal IPTp usage was 21.8%. Factors that either predispose or enable pregnant women to take optimal doses of IPTp were maternal education, being employed, being autonomous in their own healthcare, health insurance enrolment, partner education, receiving antenatal care in public health facilities, rural residence, being resident in northern geo-political zones, community literacy level and community perception of the consequences of malaria. Two significant need factors affecting optimal usage of IPTp were timing of the first antenatal care visit and sleeping under mosquito bed nets.

**Conclusion:**

Optimal usage of IPTp is low among pregnant women in Nigeria. There is a need to devise additional public health educational programs promoting IPTp usage through the formation of Advocacy, Communication and Social Mobilisation (ACSM) in every ward in all local government areas, particularly in the rural and northern parts of the country. In addition, health planners should adopt the Andersen model for assessing key determinants of IPTp usage among childbearing women in Nigeria.

## Background

Malaria in pregnancy is a specific health condition, characterised by the presence of malaria parasites in the red blood cells of the placenta. It has serious adverse effects on the health of pregnant women, the foetus and the neonate [1]. Malaria in pregnancy elevates the risks of maternal mortality or severe morbidity such as maternal anemia. It may also result in stillbirth, preterm birth, and low birth weight [1]. Based on World Health Organisation (WHO) statistics, out of the estimated 33 million pregnancies in sSA, 12 million (35%) were exposed to malaria infection in 2019 [2]. Across sSA, the prevalence of malaria in pregnancy is higher in Central and West Africa with Nigeria contributing a substantial proportion of pregnant women with malaria [2]. Apart from the rate of infection among pregnant women in the country, malaria is a key factor responsible for substantial proportions of neonatal, infant and under-five deaths [3]. Malaria also accounts for nearly 60% of hospital outpatient attendance and 30% of hospital admissions in Nigeria. This takes a heavy toll on an already inadequate healthcare system [4]. Malaria in pregnancy is thus a major public health problem requiring effective prevention strategies in all parts of Nigeria.

Across low-income countries (LIC), intermittent preventive treatment of malaria in pregnancy (IPTp) is one of the interventions widely promoted by WHO to prevent malaria [5]. Other malaria control interventions, however, include using Long-Lasting Insecticidal Nets (LLINs), Indoor Residual Spraying (IRS), and Larval Source Management [6]. Attention to IPTp emanates from increasing evidence that its use substantially reduces the risks of adverse maternal and fetal outcomes in LIC [1, 7]. Currently, in Nigeria, there is a government-approved medicine for IPTp. Pregnant women are expected to receive this medication (*SP/Fansidar)* during routine antenatal care (ANC) visits. WHO recommends at least three doses of IPTp before childbirth [5].

In Nigeria and many other sSA countries, uptake of optimal doses of IPTp (3 or more doses) by pregnant women remains low and below national targets [8–13]. For example, in the 2018 Nigeria Demographic and Health Survey (NDHS), the prevalence of IPTp optimal doses of the approved medicine was 17% among childbearing women who had given birth in the two years preceding the survey. Studies in Nigeria and elsewhere have explored factors influencing the use of IPTp, identifying some determinants of IPTp usage [8, 11, 12, 14–16]. Determinants included the type of ANC facility, health insurance enrolment, number of ANC visits, education, parity, timing of the first ANC visit, employment, place of residence, geographical region, knowledge of malaria preventive strategies, maternal age, and household wealth index [11, 17–21].

Most IPTp studies are not model or theory-based. Not using models or theories in public health research may not properly guide health planners and policymakers in malaria control programming. Models and theories are crucial to develop evidence-based health programs, particularly in the promotion of new malaria control interventions. These provide a framework for malaria program planners to build upon efforts to develop more workable initiatives [22]. One Nigerian study adapted a socio-ecological model to IPTp usage, but this was qualitative in nature and did not generate quantitative hierarchical data for the validation of the model [23]. Our study fills the knowledge gap by adapting Andersen’s behavioral model of health care use to IPTp usage in Nigeria.

Andersen model is one of the analytical behavioral models developed in the 1960s to enhance identification and measurement of diverse factors affecting family access to health care [24, 25]. Utilization of health care is explained by socio-demographic characteristics of the population, individual, households or health care resources facilitating access and need factors for care. Four types of access to health care are potential access (availability of resources or infrastructure); realized access (actual utilization); equitable and inequitable access [26]. In its current form, the model prescribes exploring contextual and individual factors affecting the use of health care [26]. Andersen model has been adapted to modelled utilization of several health care facilities. This includes the use of ANC, adolescent reproductive health care, HIV testing, contraceptive care, and long-term care and support for the elderly [27–31]. In all these studies Andersen model enhanced understanding of the underlying determinants of the use of specific health care. The objective of this study was to examine factors influencing optimal usage of IPTp in Nigeria based on the Andersen analytic framework with an additional aim to inform the 2014–2020 National Malaria Strategic Plan (NMSP) [6].

## Methods

### Design and data source

The study adopted a cross-sectional design that utilized secondary data extracted from the 2018 Nigeria Demographic and Health Survey (NDHS). NDHS was implemented by the National Population Commission (NPC) in collaboration with the National Malaria Elimination Programme (NMEP) [32]. The 2018 NDHS provided reliable information on basic demographic and health characteristics of the Nigerian population including national information about malaria infection, treatment, and control [32]. Data were analyzed with authorization from MEASURE DHS and are available online via https://dhsprogram.com/data/. The 2018 NDHS used a multi-stage sampling technique that ensured a nationally representative sample. The country was stratified into urban and rural areas after which some urban and rural areas were selected randomly. The selection was based on localities used as Enumeration Areas (EAs) in the penultimate national housing and population census. EAs served as the primary sampling unit (cluster) in the survey. Households were randomly selected in the EAs following appropriate household listing. Eligible men and women aged 15–49 were randomly selected in the different households. Comprehensive details of the methodology for the 2018 NDHS have been published elsewhere [32].

### Participants

The 2018 NDHS covered 41,821 women of reproductive age. Women who had no live birth in the past year preceding the survey (34,549 women), women who had only attended ANC in the first trimester because the government-approved drug is given in the second and third trimesters (2,359 women) and women who did not know their number of antenatal care visits (178 women) were excluded from the analysis. These criteria resulted in a weighted sample size of 4,772 women.

### Measures

The outcome variable was the usage of government-approved medicine during the last pregnancy. We categorized responses into optimal (≥ 3 doses) or otherwise (< 3 doses). This categorization was consistent with categories adopted in many studies [11, 12, 18, 19]. Explanatory variables cut across individual and community levels and were selected based on existing literature [11, 12, 14, 15, 17, 18]. These were divided into predisposing, enabling and need factors in line with the theoretical constructs of the Andersen model. Predisposing factors were age group (15–24, 25–34 and 35+), parity (primiparity – one child, multiparity – two to four children, and grand multiparity – five or more children), formal education (none, primary, secondary and higher), employment status (unemployed or employed), autonomy related to own health care (yes or no), household wealth (poorest, poorer, middle, richer and richest), place of residence (urban or rural), geo-political zone (north-central, north-east, north-west, south-east, south-south and south-west), proportion in the community who perceived malaria can cause death (low, middle and high) and proportion in the community who perceived malaria as easy to treat (low, middle and high). The community variables were generated from individual responses through aggregation at cluster level and dividing the distribution into three equal portions to derive low (0–33%), middle (34–66%), and high (67%+) categories. This method is generally used for the generation of community variables in DHS data sets [33, 34].

Enabling factors are health insurance enrolment (yes or no), source of ANC (government or private), partner education (none, primary, secondary and higher) and community literacy level (low (0–33%), middle (34–66%), and high (67%+). Need factors are the experience of the death of a child (ever or never), timing of the first ANC visit (first, second or third trimester), possession of a mosquito bed net for sleeping (yes or no), and sleeping under a mosquito bed net (yes or no). Some variables were re-coded in the study.

### Data analysis

First, frequency distribution was used to describe the socio-demographic characteristics of the women as well as the numbers who got access to IPTp. Secondly, two multilevel mixed-effects logistic regression models were fitted to examine predictors of optimal usage of IPTp. Prior to fitting the model, three mini-analyses were carried out. First, bivariate analysis using crude Odds Ratios (cOR) was carried out. Any variable showing significance at p < 0.025 was selected for inclusion in the multivariable model. This cut-off point was selected to ensure that confounding variables are eliminated. Secondly, a Variance Inflation Factor (VIF) was performed using STATA command to ensure no multicollinear independent variable was selected for the multivariable modeling. The benchmark for this test was that no variable with a VIF score of less than five should be selected for the models [35].

Thirdly, a ‘null model’ was fitted. This model did not include any explanatory variables. The essence of the null model was to ascertain whether significant variation exists in the optimal usage of IPTp across the communities, determined by the significance of the intercept of the model. Model 1 used predisposing and enabling factors to explain the inequitable use of IPTp. Model 2 used all predictor variables to examine the equitable use of IPTp in Nigeria. The analytical tool not only aligned with the theoretical position of the Andersen model but was also suitable to examine outcome predictors with hierarchical influences such as individual and community levels. This tool is widely applied in multilevel studies [33, 34]. The multilevel mixed-effects logistic model partitions influence on an outcome into fixed and random effects [36]. Fixed effects in the current study were examined using adjusted Odds Ratios (aOR), while random effects were examined using the Intra-Cluster Correlation Coefficient (ICC). ICC which ranges from zero to one indicates importance of the community factors in the overall variance of the outcome variable. Models were checked for adequacy using the Akaike Information Criterion (AIC). The model with the smallest AIC value is the best-fitted model [37]. All analyses were performed using STATA 14 [38]. Statistical significance was set at 5%.

## Results

### Socio-demographic characteristics and IPTp usage

Optimal level of IPTp usage was observed in less than a quarter of the respondents. More than half were 25–34 years old and had between two to four children (Table 1). Nearly one-third of the women had no formal education, while slightly more than two-fifths attained secondary education. With the exception of women in the poorest households, household wealth was similar among respondents (Table 1). The majority were employed, but more than half of them had no autonomy over their own health care. Nearly all women did not enroll in the national health insurance scheme.

**Table 1:** Respondents’ socio-demographic characteristics and realised usage of IPTp

Characteristic	Frequency (n = 4,772)	Percentage	Characteristic	Frequency (n = 4,772)	Percentage
**IPTp dose**			**Death of a child**		
Otherwise	3,731	78.2	Ever experienced	1,306	27.4
Optimal	1,041	21.8	Never experienced	3,466	72.6
**Maternal age group**			**Timing of first antenatal care visit**		
15–24	1,472	30.8	First trimester	1,198	25.1
25–34	2,461	51.6	s trimester	3,512	73.6
35+	839	17.6	Third trimester	62	1.3
**Parity**			**Possession of mosquito bed net**		
Primiparity (one child)	984	20.6	No	1,323	27.7
Multiparity (2–4 children)	2,306	48.3	Yes	3,449	72.3
Grand multiparity (5 + children)	1,482	31.1	**Actual sleeping under bed net**		
**Maternal education**			No	1,970	41.3
None	1,569	32.9	Yes	2,802	58.7
Primary	740	15.5	**Place of residence**		
Secondary	1,917	40.2	Urban	2,216	46.4
Higher	546	11.4	Rural	2,556	53.6
**Household wealth**			**Geo-political zone**		
Poorest	654	13.7	North-central	642	13.5
Poorer	923	19.3	North-east	822	17.2
Middle	1,044	21.9	North-west	1,539	32.2
Richer	1,092	22.9	South-east	628	13.2
Richest	1,059	22.2	South-south	415	8.7
**Work status**			South-west	726	15.2
Unemployed	1,597	33.5	**Community literacy level**		
Employed	3,175	66.5	Low	1,569	32.9
**Autonomy on own healthcare**			Middle	1,551	32.5
Not autonomous	2,724	57.1	High	1,652	34.6
Autonomous	2,048	42.9	**Proportion in community who perceived malaria is easy to treat**		
**Health insurance enrolment**			Low	1,576	33.0
Not enrolled	4,654	97.5	Middle	1,888	39.6
Enrolled	118	2.5	High	1,308	27.4
**Partner education**			**Proportion in community who perceived malaria can cause death**		
None	1,380	28.9	Low	1,462	30.6
Primary	587	12.3	Middle	1,702	35.7
Secondary	1,834	38.4	High	1,608	33.7
Higher	971	20.4			
**Source of antenatal care**					
Public health facility	3,842	80.5			
Private health facility	930	19.5			

Distribution of respondents by partner education was similar to that of maternal education, but the proportion of women with no formal education was higher than that of their partners. The majority received ANC from public health facilities and had never experienced death of a child. Most women initiated the first antenatal visit in the second trimester of pregnancy. While more than two-thirds had mosquito bed nets, a lower proportion of the women actually slept under a mosquito bed net. More than half of the respondents were rural dwellers. Women from the northern geo-political zones were dominant in the sample. Community literacy was nearly equal among the respondents. More women reside in communities with moderate proportions who believed either malaria is easy to treat or malaria could cause death (Table 1). All the explanatory variables revealed a significant independent association with optimal usage of IPTp (Table 2). All variables were thus included in the multivariate model.

**Table 2:** Bivariate analysis showing the association between the use of IPTp and the explanatory variables

Characteristic	cOR	95% CI	Characteristic	cOR	95% CI
**Maternal age group**			**Death of a child**		
15–24 ^ref^	1.00	-	Ever experienced ^ref^	1.00	-
25–34	1.59**	1.18–2.15	Never experienced	1.47**	1.29–1.68
35+	1.66**	1.33–2.07	**Timing of first antenatal care visit**		
**Parity**			First trimester ^ref^	1.00	-
Primiparity	1.00	-	Second trimester	0.64**	0.53–0.76
Multiparity	0.64**	0.53–0.76	Third trimester	0.96	0.51–1.84
Grand multiparity	0.60**	0.56–0.64	**Possession of mosquito bed net**		
**Maternal education**			No ^ref^	1.00	-
None ^ref^	1.00	-	Yes	0.79*	0.64–0.98
Primary	1.28	0.99–1.65	**Actual sleeping under bed net**		
Secondary	1.66**	1.33–2.07	No ^ref^	1.00	-
Higher	1.59**	1.18–2.15	Yes	1.28*	1.03–1.59
**Household wealth**			**Place of residence**		
**Poorest** ^**ref**^	1.00	-	Urban ^ref^	1.00	-
**Poorer**	0.89	0.66–1.19	Rural	0.52**	0.46–0.58
**Middle**	0.87	0.65–1.17	**Geo-political zone**		
**Higher**	1.56**	1.16–2.09	North-central ^ref^	1.00	-
**Highest**	1.35*	0.99–1.84	North-east	0.65**	0.54–0.79
**Work status**			North-west	0.47**	0.39–0.57
Unemployed ^ref^	1.00	-	South-east	3.11**	2.32–4.16
Employed	1.26*	1.01–1.57	South-south	1.62**	1.16–2.27
**Autonomy on own healthcare**			South-west	2.56**	2.06–3.18
Not autonomous ^ref^	1.00	-	**Community literacy level**		
Autonomous	1.89**	1.53–2.33	Low ^ref^	1.00	-
**Health insurance enrolment**			Middle	1.03	0.81–1.31
Not enrolled ^ref^	1.00	-	High	1.42**	1.12–1.80
Enrolled	2.04**	1.42–2.93	**Proportion in community who perceived malaria is easy to treat**		
**Partner education**			Low ^ref^	1.00	-
None ^ref^	1.00	-	Middle	1.29*	1.05–1.59
Primary	1.37*	1.03–1.80	High	2.15*	1.56–2.97
Secondary	2.16**	1.70–2.73	**Proportion in community who perceived malaria can cause death**		
Higher	1.60**	1.23–2.08	Low ^ref^	1.00	-
**Source of antenatal care**			Middle	1.22*	1.02–1.46
Public health facility ^ref^	1.00	-	High	1.80**	1.41–2.31
Private health facility	1.41*	1.13–1.75			

Notes: ref (reference category), *p < 0.025, **p < 0.01

### Factors influencing optimal usage of IPTp

Our fitted multilevel mixed-effects logistic regression models revealed adequate fits of the models with Model 2 showing a better fit (AIC = 5179.58) than Model 1 (AIC = 5282.46). Variance in IPTp usage across communities was high (ICC = 0.73) in the null model. These ICC values declined consistently in successive models as more variables were included. ICC-values in models 1 and 2 confirmed the significance of contextual factors.

#### Predisposing factors

In Model 1, with the exclusion of parity, all predisposing factors showed a significant influence on optimal usage of IPTp (Table 3). Inclusion of additional variables in Model 2, however, weakened the influence of maternal age, household wealth, and the proportion of women in communities who believed that malaria can cause death. As shown in Table 3, the odds of optimal IPTp usage were four times higher among women who attained higher education compared to uneducated women (aOR 4.07; 95% CI 3.47–4.79). Employed women were nearly three times more likely to utilize optimal doses of IPTp compared to unemployed women (aOR 2.59; 95% CI 2.23–3.01). While women who were autonomous with respect to their own health care compared to women who had no autonomy had higher odds of optimal usage of IPTp (aOR 2.02; 95% CI 1.03–3.96), odds were lower among rural women compared to urban women (aOR 0.44; 95% CI 0.24–0.75). While women in the northwest were less likely to utilize optimal doses of IPTp, they were more likely to utilize optimal doses of IPTp in the three southern zones. The likelihood of optimal IPTp use was lower among women who resided in communities with a high proportion of people who believed that malaria is easy to treat (aOR 0.34; 95% CI 0.20–0.60) (Table 3).

**Table 3:** Fixed and random effects on optimal IPTp usage in Nigeria

Characteristics predicting IPTp optimal use	Model 1			Model 2		
	aOR		95% CI	aOR	p-value	95% CI
**Predisposing factors:** **Maternal age group**						
15–24 ^ref^	1.00	-	-	1.00	-	-
25–34	1.49*		1.05–2.12	1.24	0.21	0.88–1.75
35+	2.12*	0.01	1.26–3.56	1.64	0.05	1.00-2.69
**Parity**						
Primiparity ^ref^	1.00	-	-	1.00	-	-
Multiparity	1.16	0.41	0.81–1.67	1.34	0.12	0.93–1.92
Grand multiparity	0.90	0.68	0.56–1.46	1.22	0.42	0.75–1.99
**Maternal education**						
None ^ref^	1.00	-	-	1.00	-	-
Primary	1.27	0.28	0.83–1.95	0.92	0.68	0.60–1.40
Secondary	2.20**	p < 0.01	1.05–2.36	1.54	0.05	0.99–2.40
Higher	2.49**	p < 0.01	1.54–4.03	4.07**	p < 0.01	3.47–4.79
**Household wealth**						
Poorest ^ref^	1.00	-	-	1.00	-	-
Poorer	1.57	0.31	0.66–3.73	0.86	0.52	0.53–1.38
Middle	1.97	0.14	0.80–4.86	0.83	0.49	0.50–1.39
Richer	3.72*	p < 0.01	2.85–4.84	0.75	0.31	0.42–1.31
Richest	4.16**	p < 0.01	3.17–5.45	1.20	0.58	0.63–2.28
**Work status**						
Unemployed ^ref^	1.00	-	-	1.00	-	-
Employed	3.29**	p < 0.01	2.67–4.06	2.59**	p < 0.01	2.23–3.01
**Autonomy on own healthcare**						
Not autonomous ^ref^	1.00	-	-	1.00	-	-
Autonomous	2.06*	0.01	1.25–3.40	2.02*	0.04	1.03–3.96
**Place of residence**						
Urban ^ref^	1.00	-	-	1.000	-	-
Rural	0.57*	0.04	0.33–0.97	0.44**	p < 0.01	0.24–0.75
**Geo-political zone**						
North-central ^ref^	1.00	-	-	1.00	-	-
North-east	1.01	0.96	0.81–1.26	1.03	0.92	0.59–1.79
North-west	0.75*	0.01	0.59–0.94	0.45*	0.01	0.24–0.82
South-east	3.72**	p < 0.01	2.86–4.84	5.05**	p < 0.01	4.06–6.27
South-south	1.81**	p < 0.01	1.38–2.37	3.39**	p < 0.01	1.66–6.89
South-west	2.01**	p < 0.01	1.57–2.57	4.37**	p < 0.01	2.15–8.89
**Proportion in community who perceived malaria is easy to treat**						
Low ^ref^	1.00	-	-	1.00	-	-
Middle	1.07	0.44	0.91–1.25	1.20	0.38	0.80–1.81
High	0.60**	p < 0.01	0.49–0.73	0.34**	p < 0.01	0.20–0.60
**Proportion in community who perceived malaria can cause death**						
Low ^ref^	1.00	-	-	1.00	-	-
Middle	1.24	0.07	0.98–1.55	1.11	0.64	0.72–1.70
High	1.35*	0.02	1.05–1.72	1.13	0.58	0.74–1.72
**Enabling factors**: **Health insurance enrolment**						
Not enrolled ^ref^	1.00	-	-	1.000	-	-
Enrolled	1.77**	p < 0.01	1.42–2.20	1.26*	0.04	1.01–1.57
**Partner education**						
None ^ref^	1.00	-	-	1.00	-	-
Primary	4.65**	p < 0.01	2.64–8.18	3.88**	p < 0.01	2.27–6.63
Secondary	5.06**	p < 0.01	2.95–8.68	4.80**	p < 0.01	2.84–8.12
Higher	3.00**	p < 0.01	1.73–5.19	3.79**	p < 0.01	2.14–6.69
**Community literacy level**						
Low ^ref^	1.00	-	-	1.00	-	-
Middle	0.92	0.84	0.82–1.18	1.04	0.86	0.66–1.64
High	1.29*	0.02	1.05–1.59	1.75*	0.03	1.06–2.90
**Source of antenatal care**						
Public health facility ^ref^	1.00	-	-	1.00	-	-
Private health facility	1.91**	p < 0.01	1.29–2.82	2.26*	0.02	1.12–4.59
**Need factors**: **Death of a child**						
Ever experienced ^ref^				1.00	-	-
Never experienced				1.13	0.45	0.83–1.54
**Timing of first antenatal care contact**						
First trimester ^ref^				1.00	-	-
Second trimester				1.47**	p < 0.01	1.29–1.68
Third trimester				0.53*	0.04	0.29–0.96
**Possession of mosquito bed net**						
No ^ref^				1.00	-	-
Yes				1.01	0.96	0.67–1.52
**Actual sleeping under mosquito bed net**						
No ^ref^				1.00	-	-
Yes				0.72*	0.05	0.52–0.99
AIC	5282.46			5179.58		
ICC	0.56 (55.7%)			0.51 (50.6%)		

Notes: ref (reference category), *p < 0.05, **p < 0.01

#### Enabling factors

All enabling factors revealed significant influence on the likelihood of optimal usage of IPTp in Model 1. These significances were not altered in the subsequent model. Women who were enrolled in health insurance were more likely to utilize optimal doses of IPTp compared to not enrolled women (aOR 1.26; 95% CI 1.01–1.57) (Table 3). Women whose husbands had improved educational attainments had higher odds of IPTp usage. Women who resided in communities with high literacy levels were more likely to utilize optimal IPTp compared to those in communities with low literacy levels (aOR 1.75; 95% CI 1.06–2.90). Also, women who received antenatal care from private health facilities were more than twice more likely to take optimal doses of IPTp compared to women who received antenatal care from public health facilities (aOR 2.26; 95% CI 1.12–4.59).

#### Need factors

Timing of the first ANC visit and women’s actual sleeping under mosquito bed nets were the two significant need factors. While the odds of optimal usage of IPTp were higher among women who initiated ANC visits in the second trimester compared to those who started in the first trimester (aOR 1.47; 95% CI 1.29–1.68), the odds were lower among women who initiated first ANC contact in the third trimester (aOR 0.53; 95% CI 0.29–0.96) (Table 3). Women who actually slept under mosquito bed nets were less likely to utilize optimal doses of IPTp (aOR 0.72; 95% CI 0.52–0.99).

## Discussion

Low use of optimal doses of IPTp was found among women with recent births in Nigeria. This is consistent with evidence provided in government reports and several hospitals or population-based studies [8, 10, 11, 16]. This low prevalence, however, deviates widely from other studies and may be due to lack of uniformity in the categorization of IPTp use in the literature [12, 14, 15, 18, 19]. Contrary to our study, many studies grouped IPTp use into three or more categories, undermining the opportunity to observe the extent of optimal use [1, 15]. Also, a study derived IPTp prevalence from women who gave birth in the last two or three years, while our study focused only on women giving birth during the last year [21].

One key take away from the low level of optimal IPTp use is that the NMSP needs to be rejuvenated in Nigeria to achieve its objectives [6]. Objective one of that strategy seeks to ensure that at least 80% of targeted population utilizes appropriate preventive measures by 2020. Since we studied only women who had at least three antenatal contacts, our findings suggest that some women experience barriers in accessing IPTp. This may not only lead to poor treatment of malaria infections but also suggests missed opportunities to provide pregnant women with IPTp in the country. Furthermore, low levels of optimal IPTp use undermine the third objective of NMSP to treat all individuals with confirmed malaria with effective antimalaria drugs by 2020. Reasons for low use may probably be due to either poor knowledge of IPTp by pregnant women or the existence of health system barriers such as stock out of IPTp and poor training of providers [13, 20]. The FMoH may need to devise additional public health educational programs that promote IPTp use. One way to achieve this is to ensure that Advocacy, Communication and Social Mobilisation (ACSM) core groups suggested in the plan are constituted in every Local Government Area. ACSMs will further engage the community and distribute more Information, Education and Communication (IEC) materials. Health sector barriers may also be identified and addressed through timely monitoring and evaluation activities.

Predisposing factors such as poor maternal education, rural residence and lack of autonomy with respect to women’s own health care hindered optimal use of IPTp in line with other studies [18, 21]. Improved education and female autonomy were significant sources of empowerment that helped women not only to understand health needs but also to access health care. Urban residence often promotes the use of health care due to more availability of health facilities in urban settings. Consistent with similar studies, unemployment, and northern residence reduced use of optimal IPTp [12, 14, 19]. Unemployed women may face particular difficulties such as sourcing money for transport to facilities. One likely cause of lower use in a mainly Islamic Northern Nigeria where female health care providers are not easily available in facilities.

Enabling and need factors such as source of ANC, health insurance enrolment, partner education, timing of first antenatal contact, and actual sleeping under insecticidal treated bed nets enabled optimal use of IPTp in agreement with other studies [11, 12, 14, 15, 17, 19, 20]. Contrary to a recent study in Tanzania we found higher use of IPTp in private health facilities compared to public ones [11]. In Nigeria, maternal and child health care is mostly provided in government health facilities. The few pregnant women who use private facilities are often well-educated and wealthier and such facilities are likely to possess sufficient drugs and resources for effective treatment. In contrast, public facilities sometimes experience stock out of drugs. Early antenatal contact is crucial for prompt detection and effective treatment of malaria. In addition, IPTp has to be given at specified weeks of gestation, presupposing that pregnant women adhere to the prescribed ANC visits [5].

Our study has a few drawbacks. Data are self-reported and not subjected to additional scrutiny. Being a further analysis of secondary data, we had no control over data collection procedures. We assumed, however, that NDHS datasets are trustworthy, having been collected through standard procedures. Also, most variables in the study were used as captured in the NDHS. There was no opportunity of using feedback from the pilot test to modify the measures. We only focused on the use of IPTp, while other types of treatment exist which may lead to non-use of IPTp. Usage of IPTp may not therefore accurately mirror malaria control strategies during pregnancy in the country. The nature of our study is cross-sectional and thus does not permit any claim of cause-effect relations. Using the term ‘influence’ in the multivariable analysis thus established a strong association between the variables, but not necessarily causality.

## Conclusion

This study using secondary data from NDHS and based on the Andersen behavioral model of health care use, examined factors influencing optimal use of IPTp in Nigeria. A number of predisposing, enabling, and need factors underlying IPTp optimal use were identified. Low usage of optimal IPTp may be improved through additional public health educational initiatives promoting IPTp. In addition, health planners in the country particularly the FMoH should adopt the Andersen model not only for assessing the underlying factors of IPTp use among reproductive-age women but also for the evaluation of health care interventions in the country.

## Data Availability

Secondary data analysed could be accessed online at https://dhsprogram.com/data/.

## Declarations

### Compliance with guidelines

I confirm that all methods were performed in accordance with the relevant guidelines.

## Abbreviations

ACSM: Advocacy, Communication, and Social Mobilisation
ANC: Antenatal care
FMoH: Federal Ministry of Health
IPTp: Intermittent preventive treatment of malaria in pregnancy
IRS: Indoor Residual Spraying
LLINs: Long Lasting Insecticidal Treated Nets
NDHS: Nigeria Demographic and Health Survey
NPC: National Population Commission
WHO: World Health Organization
NMSP: National Malaria Strategic Plan
